# Immunogenicity and safety of an intradermal ChAdOx1 nCoV-19 boost in a healthy population

**DOI:** 10.1038/s41541-022-00475-z

**Published:** 2022-05-13

**Authors:** Nawamin Pinpathomrat, Porntip Intapiboon, Purilap Seepathomnarong, Jomkwan Ongarj, Ratchanon Sophonmanee, Jariya Hengprakop, Smonrapat Surasombatpattana, Supattra Uppanisakorn, Surakameth Mahasirimongkol, Waritta Sawaengdee, Supaporn Phumiamorn, Sompong Sapsutthipas, Chanon Kongkamol, Thammasin Ingviya, Pasuree Sangsupawanich, Sarunyou Chusri

**Affiliations:** 1 Department of Biomedical Sciences and Biomedical Engineering, Faculty of Medicine, Prince of Songkla University, Songkhla, Thailand; 2 Department of Internal Medicine, Faculty of Medicine, Prince of Songkla University, Songkhla, Thailand; 3 Department of Pathology, Faculty of Medicine, Prince of Songkla University, Songkhla, Thailand; 4 Clinical Research Center, Faculty of Medicine, Prince of Songkla University, Songkhla, Thailand; 5 Department of Medical Science, Ministry of Public Health, Nonthaburi, Thailand; 6 Institute of Biological Products, Department of Medical Sciences, Ministry of Public Health, Nonthaburi, Thailand; 7 Division of Digital Innovation and Data Analytics, Faculty of Medicine, Prince of Songkla University, Songkhla, Thailand

**Keywords:** Peptide vaccines, Inactivated vaccines, Viral infection, Antibodies, Viral infection

## Abstract

Severe acute respiratory syndrome coronavirus 2 (SARS-CoV-2) has caused a global pandemic. Two doses of an inactivated SARS-CoV-2 vaccine (CoronaVac) have been shown to be insufficient to protect against variants of concern (VOCs), while viral vector vaccines remain protective against the infection. Herein, we conducted a preliminary study to evaluate the safety and immunity in an adult population who received the conventional 2 dosage-regimen of inactivated SARS-CoV-2 vaccine; with an additional intradermal ChAdOx1 nCoV-19 reciprocal dosage (1:5). An Intramuscular ChAdOx1 nCoV-19 booster was also included as a control. Immediate and delayed local reactions were frequently observed in the fractional intradermal boost, but systemic side effects were significantly decreased compared to the conventional intramuscular boost. The anti-RBD-IgG levels, the neutralising function against delta variants, and T cell responses were significantly increased after boosting via both routes. Interestingly, the shorter interval elicited higher immunogenicity compared to the extended interval. Taken together, a reciprocal dosage of intradermal ChAdOx1 nCoV-19 booster reduces systemic adverse reactions and enhances non inferiority humoral and cellular immune responses compared to a full dose of intramuscular boosting. These findings provide for an effective vaccine management during the shortages of vaccine supply.

## Introduction

During the pandemic of COVID-19, caused by SARS-CoV-2, there were more than 372 million infected patients and more than 5.6 million deaths worldwide; as of the end of January 2022. In addition to the substantial mortality due to COVID-19, this pandemic has consumed medical resources at an alarming rate. The need for a truly mass immunisation programme will be considered^1^. The massive vaccination campaign has posed significant ethical and executional challenges to us as vaccine developers, as well as to the respective principal investigators, safety review boards and regulators^2^.

The viral mutations and variants have emerged and are problematic. The B.1.617.2 (Delta) variants, which was first identified in India, then Great Britain, the United States and in Thailand is one such variant of concern^3^. The delta variant is characterised by its receptor-binding protein mutations, which result in its capacity to increase its replication and its transmission rate^4^. Both the limitation of the availability of vaccines and the data on the effectiveness of Covid-19 vaccines against this variant has been limited.

Recently, the effectiveness of two vaccines against the delta variant of SARS-CoV-2 have been examined; these being: an mRNA-based vaccine (BNT162b2; tozinameran) produced by Pfizer Inc and BioNTech SE and a replication-deficient simian adenovirus vector ChAdOx1 nCoV-19 (Vaxzevria) from Oxford University and AstraZeneca. The results revealed modest differences in effectiveness being noted with the delta variant as compared to the alpha variant. The effectiveness against the disease from two doses of the BNT162b2 vaccine was 93.7% (95% CI, 91.6 to 95.3), while with the ChAdOx1 nCoV-19 vaccine it was 74.5% (95% CI, 68.4 to 79.4)^5^. In addition, the estimated neutralisation capacity of the Pfizer–BioNTech vaccine against variant delta was 5.8-folds reduced^6^. Neutralising activity against the delta strain induced by CoronaVac (Sinovac) vaccination was lower when compared to natural infection^7^. However, the efficacy data in Thailand is still insufficient, with the vast majority of vaccination of an inactivated SARS-CoV-2 vaccine (CoronaVac).

Even though vaccination is one of the cornerstones in controlling COVID-19 outbreaks, reducing mortality, and protecting population health, the reports of COVID-19 infection among vaccinated HCWs are on the rise^8^. Several reports have demonstrated a declined immunity against SARS-CoV-2 in long-term cohorts after vaccination^9–11^. Thus, the existing immunity of the originally conventional vaccination is not sufficient to protect against the potential emerging variants of the concerning strains of SARS-CoV-2^10,12,13^. There have been several strategies to improve the prevention of infection; including, mixing and switching vaccinations during the shortage of supply; chemoprophylaxis and boosting the immunity against this virus^14^. The strategies for vaccine prioritisation and mass dispensing were not suitable in the countries with an insufficient volume of vaccination supply, and for those with limited types of vaccines^14^. The data on chemoprophylaxis with ivermectin is still unclear^15,16^. Herein, the purpose of this current study was to focus on boosting immunity in addition to the conventional regimen of the existing vaccine; an inactivated SARS-CoV-2 vaccine (CoronaVac).

On the basis of insufficient data on boosting dosages, a lower volume of ChAdOx1 nCoV-19 (Oxford-AstraZeneca) was preferred; in regard to the minimisation of dose-dependent adverse reactions. Then the appropriate route of administration via intradermal was suggested. The efficacy of fractional intradermal vaccination in comparison with full doses has been conducted for the following pathogens: influenza virus, rabies virus, poliovirus (PV), hepatitis B virus (HBV) and hepatitis A virus (HAV)^17,18^. In a TB vaccine study, an intradermal boost of a viral vector vaccine, after an attenuated pathogen prime, showed superior protection and enhanced strong cellular and humoral immune responses^19,20^. Intradermal vaccination of the Chimpanzee Adenoviral (ChAd) vectored vaccine has also been conducted. AdCh63 ME-TRAP, non-replicating viral vectors (ChAd63) expressing the insert (ME-TRAP)^21^, was administered intradermally instead of intramuscularly (IM). In this study, a 1:5 dose (1 × 10^5^ viral particles) of intradermal vaccination-induced good immune responses, which were comparable with a full dose of IM^22^. Recent studies showed intradermal vaccination with fractional doses (1:5 and 1:10) of mRNA-1273 (Moderna) enhanced similar antibody responses compared to full dose IM vaccination^23^. In people with past infection, a high level of anti-spike IgG was detected after mRNA vaccine BNT162b2 (Pfizer–BioNTech) vaccination within a 4–8-week interval; additionally, longer intervals, 8–12 and >12 weeks provided higher humoral responses^24^. Therefore, ChAdOx1 nCoV-19 are suitable to be used as a booster dose for our study.

Herein, we conduct a preliminary study to evaluate the immunity of the adult population who received the conventional two dosage regimen of inactivated SARS-CoV-2 vaccine (CoronaVac) with an additional intradermal ChAdOx1 nCoV-19 reciprocal dosage in 4–8 and 8–12-week intervals.

## Results

### Study participants

**Fig. 1:**
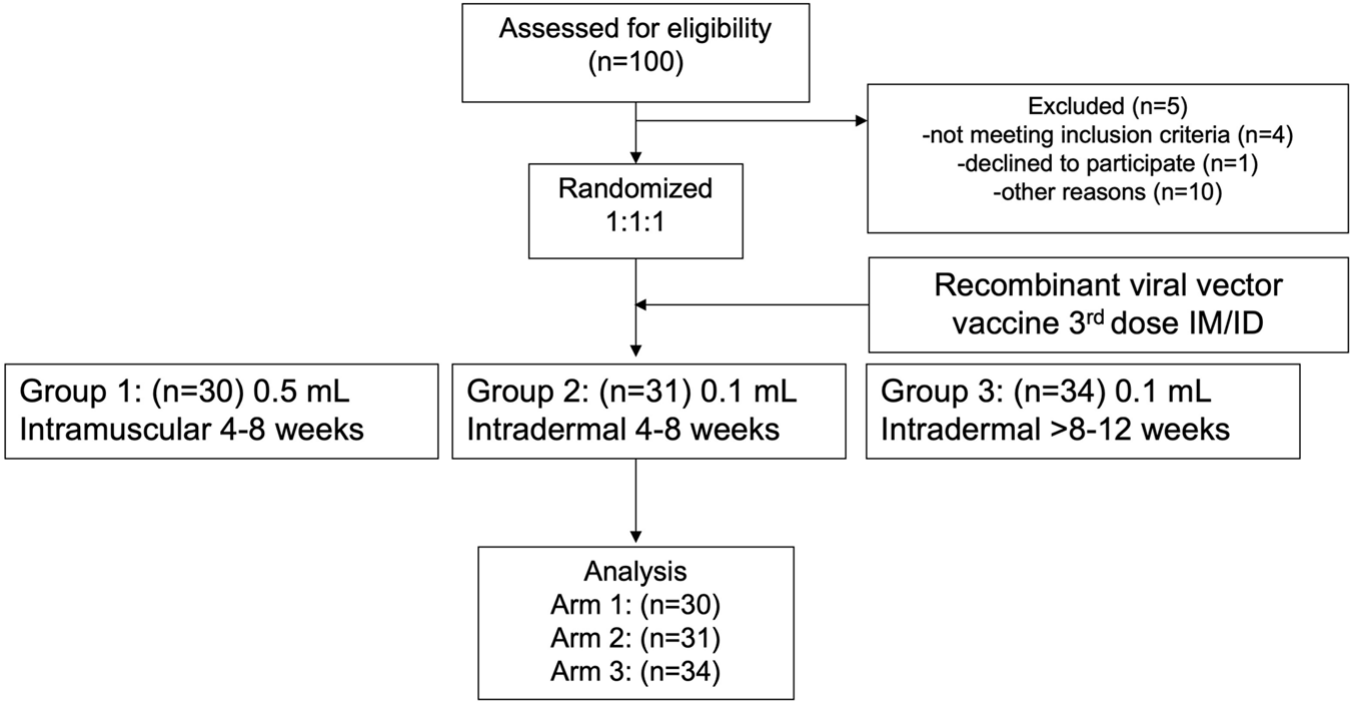
CONSORT chart of study design and volunteer recruitment. Healthy volunteers who had been vaccinated with two doses of CoronaVac were recruited into the study. 94 participants were enrolled and randomised into 3 study groups. Group 1 participants were received a full dose of ChAdOx1 nCoV-19 intramuscularly. Group 2 and Group 3 volunteers were vaccinated intradermally with a fractional dose of the viral vector vaccine. The interval between completed primary series of CoronaVac and the booster was 4–8 weeks in Group 1 and 2 but was > 8–12 weeks in Group 3. ID intradermal, IM intramuscular.

The demographics of the study participants are shown in Table 1. Healthy adults aged 18–60 years (*n* = 95), who had received a two dosage regimen of inactivated SARS-CoV-2 vaccine for more than 4 weeks were recruited for this study. The median participant age was 36 years old, and there were no differences between the treated groups. The interval between the first and second dose of the inactivated vaccine was 21 days, and the median time to booster dose (third dose) was 53 days. The study outline is shown in Fig. 1. In total 61 participants, who were within the interval of 4–8 weeks after completing vaccination, were randomised to receive either; 5 × 10^10^ viral particles of ChAdOx1 nCoV-19 intramuscularly (Group 1) or one-fifth of the viral vector vaccine intradermally (Group2). For the interval of >8–12 weeks, 34 participants consented to receive the same injection as group2 (Group3). Blood samples were collected on the day of vaccination, then at 14 and 28 days after vaccination for immunological analysis.

**Table 1:** Baseline characteristics of the intramuscular and intradermal. boosted ChAdOx1 nCoV-19 (Oxford-AstraZeneca; AZ) in the tested groups.

Baseline characteristic	Total	AZ IM	AZ ID	AZ ID	*P* value
		(4–8 wk)	(4–8 wk)	(8–12 wk)	
	*n* = 95 (%)	*n* = 30 (%)	*n* = 31 (%)	*n* = 34 (%)	
Female	74 (77.9)	25 (83.3)	25 (80.6)	24 (70.6)	0.426
Median age, year (IQR)	36	35.0 (28,43.2)	33.0 (30.5,41.5)	38.0 (33.2,44.8)	0.253
Median vaccine duration, day (IQR)	21	21 (21,26)	21 (20,28)	21 (17,21)	0.006
Median time of vaccine booster, day (IQR)	53	47 (45,52)	51 (44,52)	80.5 (69.8,82)	<0.001

### Immediate and delayed adverse events

**Fig. 2:**
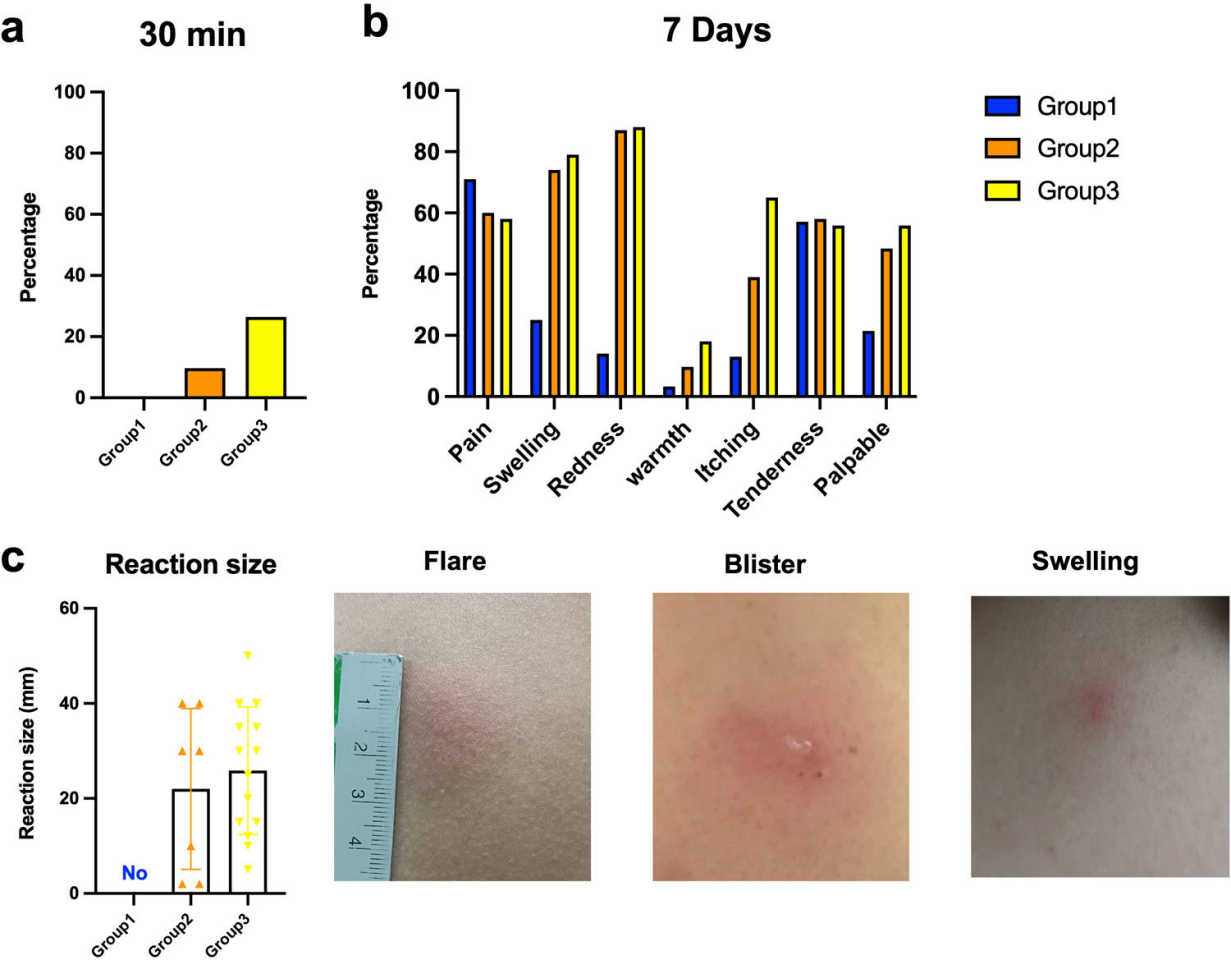
Solicited local adverse reactions at 30 min and 7 days after boosting. Full dose of intramuscular (Group 1, blue) and one in five doses of intradermal viral vector vaccines were given 4–8 weeks after completed vaccination with two doses of inactivated SARS-CoV-2 (Group2, orange). Some of the vaccinated individuals were boosted with a fractional dose of intradermal viral vector 8–12 weeks after completing two doses of the inactivated vaccine (Group3, yellow). **a** The immediate local reactions were observed within 30 min after injection. **b** Seven days after boosting, local adverse events were recorded to compare between booster groups. **c** The margins of local reaction size were measured and recorded as millimetres (mm).

The adverse event analysis is illustrated in Table 2. The most commonly reported immediate adverse events were local reactions (22.1%): all occurred in the intradermal groups (0% in group1, 22.6% in group2 and 41.2% in group3) (Fig. 2a). The difference between IM and ID booster given at 4–8-week intervals was significant, *p* = 0.011), all reactions were less than 5 cm in diameter and spontaneously resolved within 1-week post booster (Fig. 2c). Neither sign of anaphylaxis nor moderate to severe, immediate adverse event were observed.

**Table 2:** Adverse events of the intramuscular (IM) and intradermal (ID) boosted ChAdOx1 nCoV-19 (Oxford-AstraZeneca; AZ) in the tested groups.

		AZ IM (4–8 wk)	AZ ID (4–8 wk)	AZ ID (8–12 wk)	
Adverse events	Total	Group1	Group2	Group3	*P* value
	*n* = 95 (%)	*n* = 30 (%)	*n* = 31 (%)	*n* = 34 (%)	
Immediate (30 min)	21 (22.1)	0 (0)	7 (22.6)	14 (41.2)	0.011
Delayed (7 days)	71 (74.7)	20 (66.7)	25 (80.6)	26 (76.5)	0.081
Pain	53 (55.8)	20 (66.7)	15 (48.8)	18 (52.9)	0.324
Swelling	57 (60.0)	7 (23.3)	23 (74.2)	27 (79.4)	<0.001
Erythema	61 (64.2)	4 (13.3)	27 (87.1)	30 (88.2)	<0.001
Nodule	40 (42.1)	6 (20.0)	15 (48.4)	19 (55.9)	0.01
Local reactions treatment					0.405
Grade 1	85 (97.7)	26 (100)	29 (100)	30 (93.8)	
Grade 2	2 (2.3)	0 (0)	0 (0)	2 (6.2)	
Systemic reactions	42 (44.2)	19 (63.3)	12 (38.7)	11 (32.4)	0.096
Fever	11 (11.6)	8 (26.7)	1 (3.2)	2 (5.9)	0.012
Chill	20 (21.1)	12 (40)	5 (16.1)	3 (8.8)	0.073
Fatigue	24 (25.3)	11 (36.7)	5 (16.1)	8 (23.5)	0.126
Headache	22 (23.2)	11 (36.7)	6 (19.4)	5 (14.7)	0.222
Myalgia	31 (32.6)	16 (53.3)	9 (29)	6 (17.6)	0.095
Dyspnea	4 (4.2)	3 (10)	1 (3.2)	0 (0)	0.354
Joint pain	4 (4.2)	3 (10)	1 (3.2)	0 (0)	0.354
Vomiting	1 (1.1)	1 (3.3)	0 (0)	0 (0)	0.492
Systemic reactions treatment					0.174
Grade 1	14 (33.3)	2 (10.5)	4 (33.3)	8 (72.7)	
Grade 2	28 (66.7)	17 (89.5)	8 (66.7)	3 (27.3)	

*P* value refers comparisons between Group1 and Group2 which were determined using Chi’s square test.

**Fig. 3:**
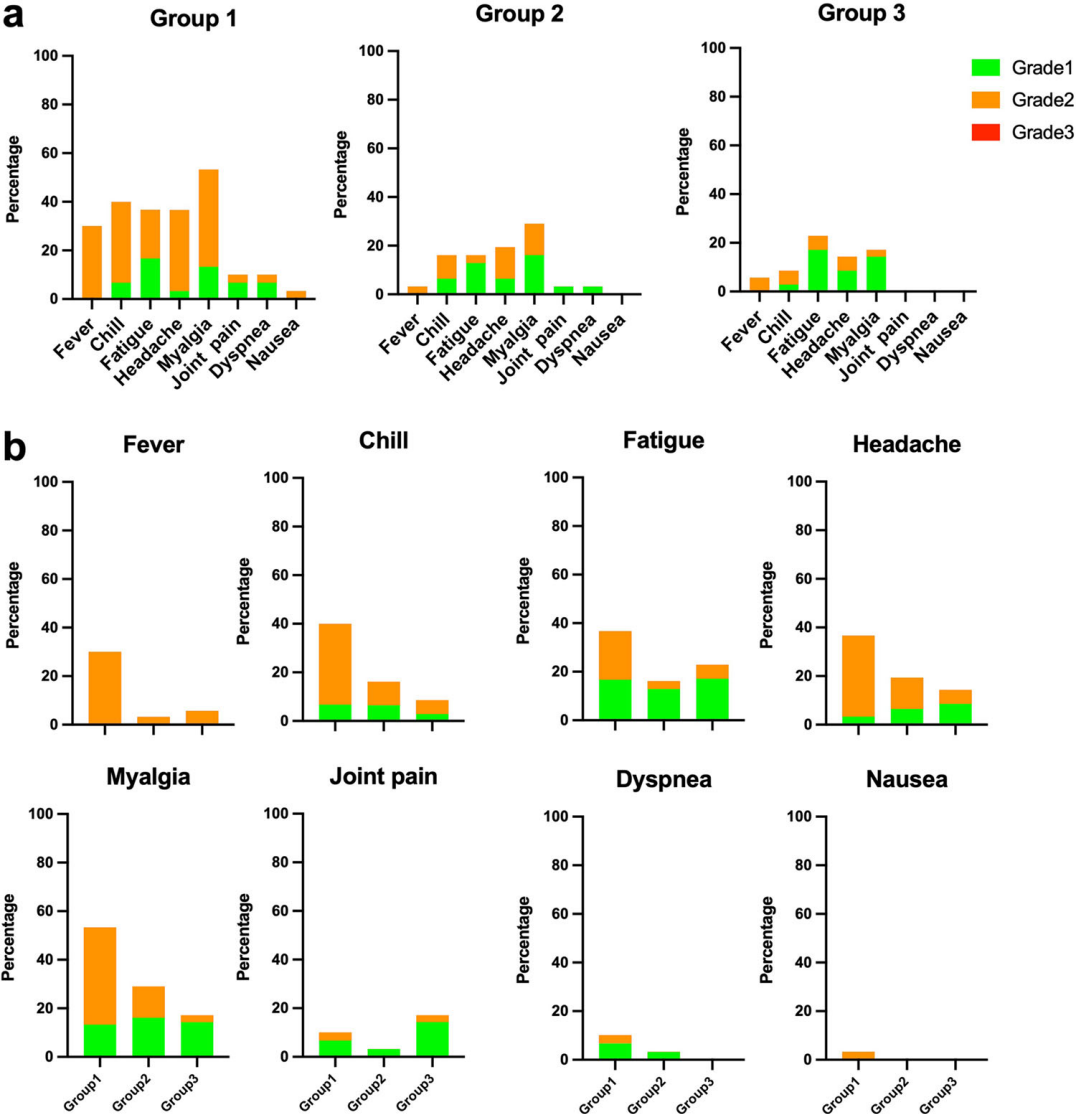
Solicited systemic adverse reactions in 7 days after boosting. One in five dosages of viral vector vaccine was delivered intradermally 4–8 weeks (Group2) and 8–12 weeks (Group3) after 2 doses of inactivated SARS-CoV-2 vaccines. Intramuscular viral vector boost, with a 4–8-week interval after the last vaccination (Group 1), was also included as controls. **a** The systemic adverse events were graded as per medical requirements and presented as percentages. The self-limited, systemic reactions were grade 1 (green). The reactions requiring medications were grade 2 (orange), and those needing medical attention were grade 3 (red). No grade 3 reactions were observed. **b** Systemic reactions were plotted separately to compare the three vaccinated groups.

Regarding the delayed local reactions, 74.7% had at least one local reaction. The prevalence of swelling, erythema and nodule were more common in the intradermal groups (Fig. 2b). The prevalence of delayed, systemic reactions was 44.2%. Interestingly, we observed a higher rate in the intramuscular group compared with the intradermal subgroup (63.3 vs. 38.7%, *p* = 0.096); especially, fever, chill, and myalgia (Fig. 3). In addition, most of the participants (89.5%) in the intramuscular group required symptomatic treatment (Grade 2); however, no patient required a doctor’s attention (Fig. 3). At 4 weeks post booster, no serious adverse effects were reported.

### SARS-CoV-2 anti-RBD antibody responses; induced by intradermal and intramuscular ChAdOx1 nCoV-19 booster

**Fig. 4:**
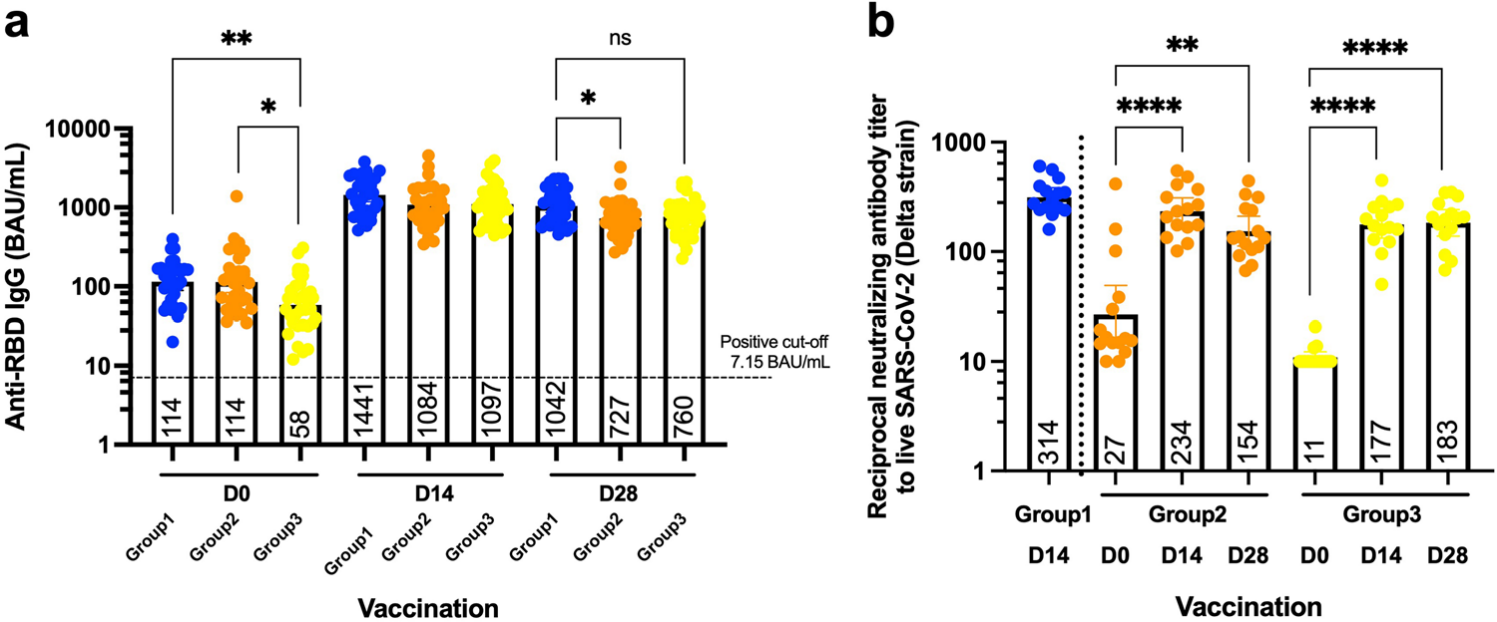
Antibody responses and neutralising function after boosting. The fractional dose of the viral vector vaccine was delivered intradermally, 4–8 weeks (Group2) and 8–12 weeks (Group3) after two doses of inactivated SARS-CoV-2 vaccines. Intramuscular viral vector boosting was also given at 4–8 weeks after the last vaccination (Group 1). The blood samples were collected before (D0) and after the booster dose for 14, 28 days (D14, D28). **a** Serum samples were analyzed using CMIA to measure anti-RBD IgG. **b** Neutralising function of antibodies against the delta variant were tested using PRNT. Each symbol represents one participant, and the number is the geometric mean with 95% CI (*n* = 30–34 volunteers). Statistical significance was determined using Kruskal–Wallis test, with Dunn’s multiple comparisons test between vaccinated groups (a) and between time points (**b**). *p ≤ 0.05, **p ≤ 0.01, ****p ≤ 0.0001, ns non significance.

The level of anti-RBD IgG has been suggested to be correlated with vaccine efficacy^25^. Before and after booster doses of intramuscular and intradermal viral vectors, blood samples were collected, and sera were analysed to observe antibody responses. The anti-RBD IgG level after completion of two doses of the inactivated vaccine was measured as a baseline. The antibody level was significantly increased 14 days after a conventional booster of a full dose viral vector was boosted intramuscularly (Fig. 4a). Intradermal boost using 1 in 5 of the standard dosage enhanced comparable IgG responses with the conventional boosting. The intervals of 4–8 weeks and 8–12 weeks after completing two doses of inactivated vaccines, until the third dose, provided similar responses after the fractional dose of an intradermal booster (Fig. 4a). After 28 days of receiving the booster, the antigen-specific IgG remained significantly higher compared to the baseline. The antibody level between the three boosted groups were comparable. The magnitude of the antibody responses in D28 was slightly decreased compared to D14; however, the differences were not statistically significant (Fig. 4a).

### Plaque reduction neutralisation test (PRNT)

The neutralising activity of antibodies is crucial for observing their protective functions against the variants of concern^6,7^. The serum samples were diluted before being tested with the live virus. The ability to reduce 50 percent of the infective units were recorded and presented. After two doses of the inactivated vaccines for more than 4 weeks, the neutralising function of the obtained antibodies were very low (Fig. 4b). After 14 days, of an intradermal boost with the fractional dose of viral vector vaccine, the neutralising functions against the delta variant were significantly improved in both the 4–8-week interval (*p* = 0.0020) and 8–12 week interval (*p* = 0.0002) (Fig. 4b). The neutralising function continued to rise after 28 days post boosting in the latter interval (*p* < 0.0001), but not in the early one (*p* = 0.4077) (Fig. 4b). The neutralising titres in the intradermal groups were not significantly different compared to the intramuscular controls (Fig. 4b).

### T cell responses induced by intradermal and intramuscular ChAdOx1 nCoV-19 booster

**Fig. 5:**
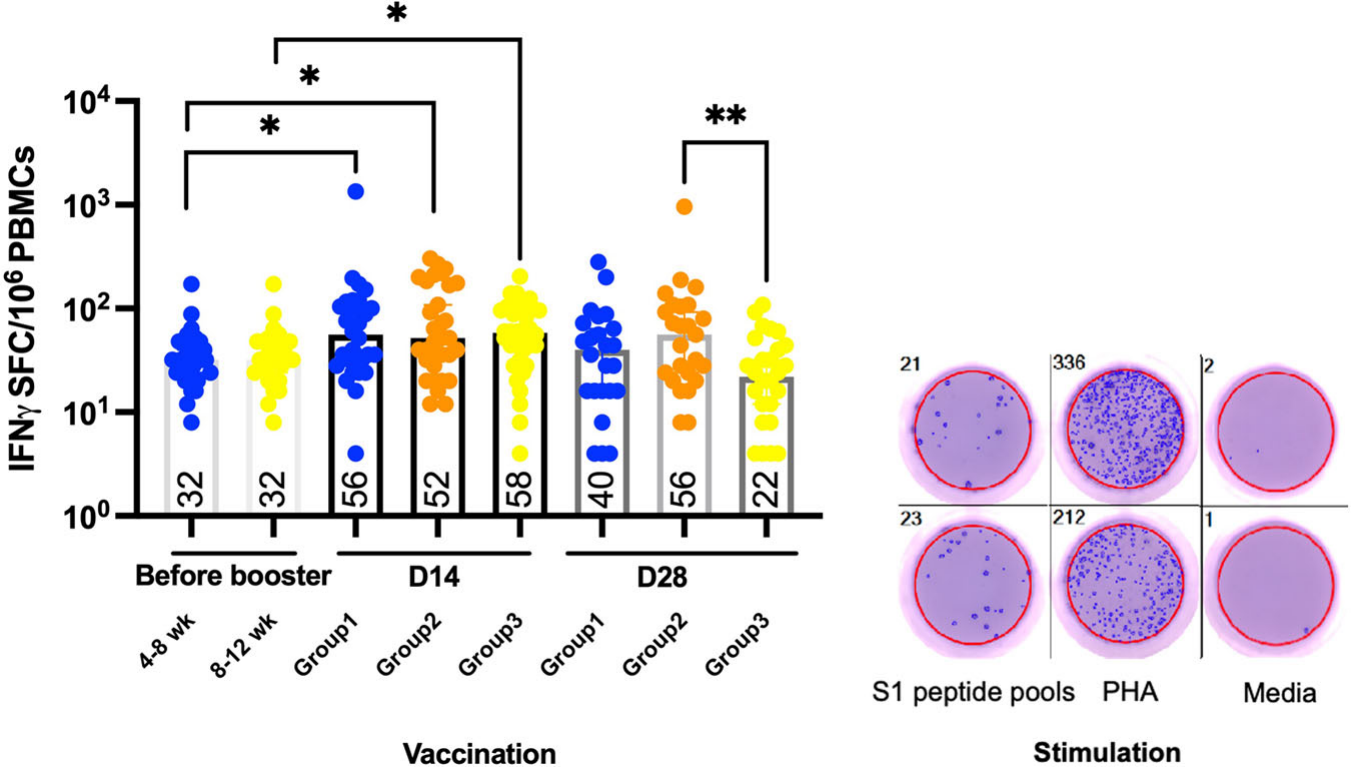
T cell responses after the booster. All volunteers were previously vaccinated with two doses of inactivated SARS-CoV-2 vaccines. After 4–8 weeks (Group2) and 8–12 weeks (Group3), the fractional dose of a viral vector vaccine was delivered intradermally; as a booster dose. A full dose of intramuscular viral vector booster was also given at 4–8 weeks after their last vaccination (Group 1). Blood was taken before (D0) and after the booster dose for 14, 28 days (D14, D28). The blood samples were processed to obtain PBMCs. The fresh PBMCs were stimulated with S1 peptide pools before measuring IFN-γ secreted cells, using ELISpot. PHA, was included as a positive control. Media was used in unstimulated controls. Each symbol represents one participant, and the number is the median of each group with 95% CI (*n* = 30–34 volunteers). Statistical significance was determined using Kruskal–Wallis test, with Dunn’s multiple comparisons test between vaccinated groups. **p ≤ 0.01.

Antibodies help to protect against the virus from infecting the cells, while T cells play an important role to clear the infected cells^26^. As far as T cell responses are concerned, PBMCs were collected pre and post boosting with the viral vector vaccines. S1 peptide pools were used to re-stimulate the cells ex vivo before the IFN-γ producing cells were stained. Pre-existing responses before boosting were observed in completed inactivated SARS-CoV-2 vaccination (Fig. 5). After 14 days from the third dose, the cytokine secreting cells were significantly enhanced after the intramuscular boost, with the full dosage and the intradermal boost, and with a fractional dose of the adenoviral vector vaccine at a 4–8-week interval (*p* = 0.0151 and *p* = 0.0102, respectively) (Fig. 5). For the longer interval, the response was also increased when compared to before boosting (*p* = 0.0129) (Fig. 5). After 28 days of boosting, T cells responses were also enhanced; however, they were not significant compared to the baseline responses of their own intervals. Lower T cell responses were observed in the longer interval of the intradermal boosted group compared to the 4–8-week interval (*p* = 0.0093) (Fig. 5). No other differences were found between the boosted groups at both 14 days and 28 days after the boosting (Fig. 5).

### Antigen-specific cytokine production of T cells after the booster

**Fig. 6:**
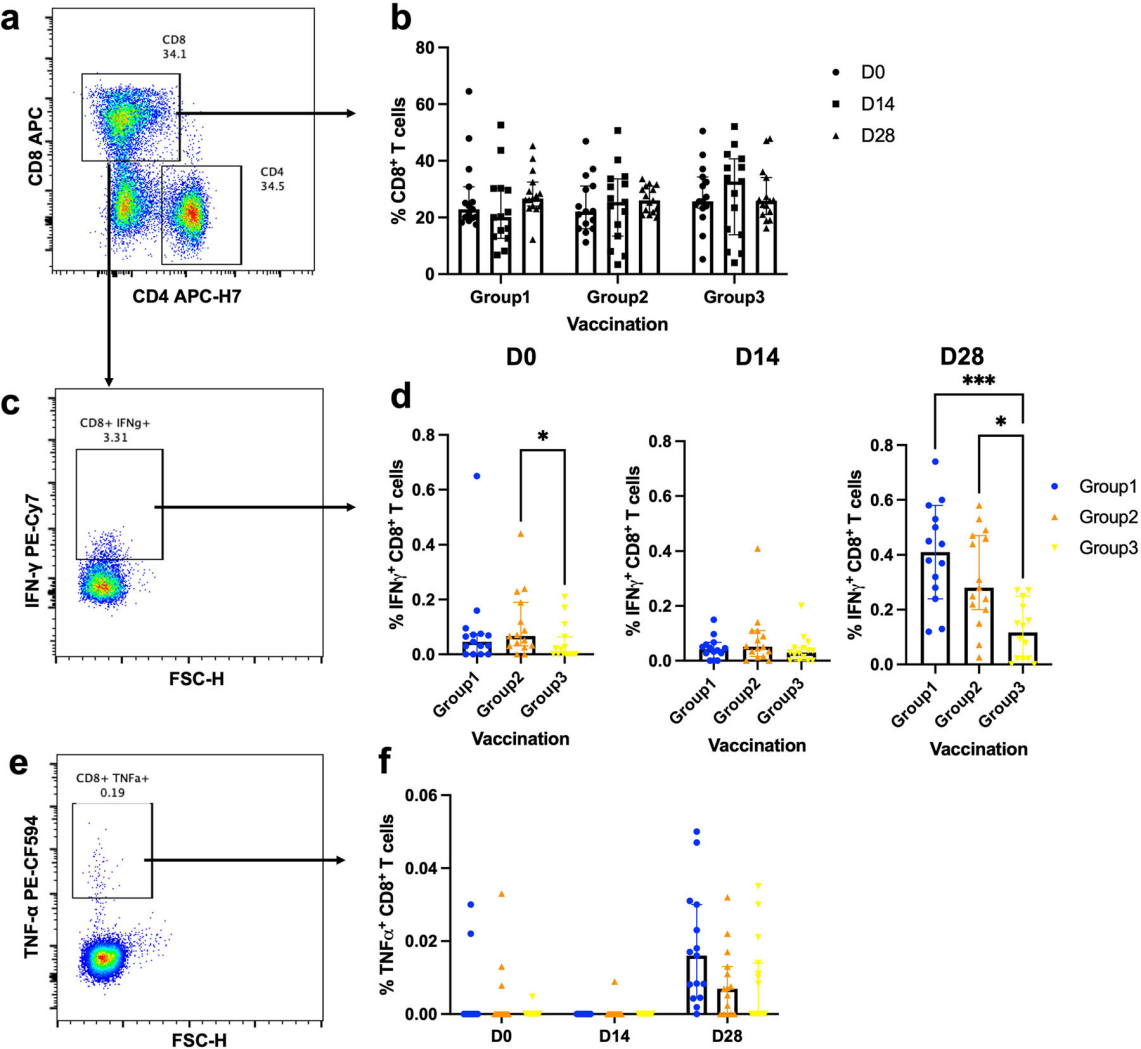
Effector cytokine production of S1-specific CD8 + T cells after boosting. All volunteers were previously vaccinated with two doses of inactivated SARS-CoV-2 vaccines. After 4–8 weeks (Group2) and 8–12 weeks (Group3), the fractional dose of a viral vector vaccine booster was delivered intradermally. A full dose of intramuscular viral vector booster was also given at 4–8 weeks as a control (Group 1). Blood was taken before (D0) and after the booster dose for 14, 28 days (D14, D28). The blood samples were processed to obtain PBMCs. The frozen PBMCs were thawed and stimulated with S1 peptide pools. The cells were stained and analysed using flow cytometry. **a** Representative flow plot shows CD8 + T cell and CD4 + T cell populations. **b** Percentage of CD8 + T cells before and after the booster dose. **c** CD8 + T cells were then selected for S1-specific IFN-γ producing cells. **d** Percentage of S1-specific IFN-γ producing CD8 + T cell responses at D0, D14, and D28. **e** Representative flow plot shows CD8 + T cells producing S1-specific TNF-α. **f** S1-specific TNF-α producing CD8 + T cells. Each symbol represents one participant presenting as a median with 95% CI (*n* = 30–34 volunteers). Statistical significance was determined using Kruskal–Wallis test, with Dunn’s multiple comparisons test between vaccinated groups. *p ≤ 0.05; **p ≤ 0.01; ***p ≤ 0.001.

**Fig. 7:**
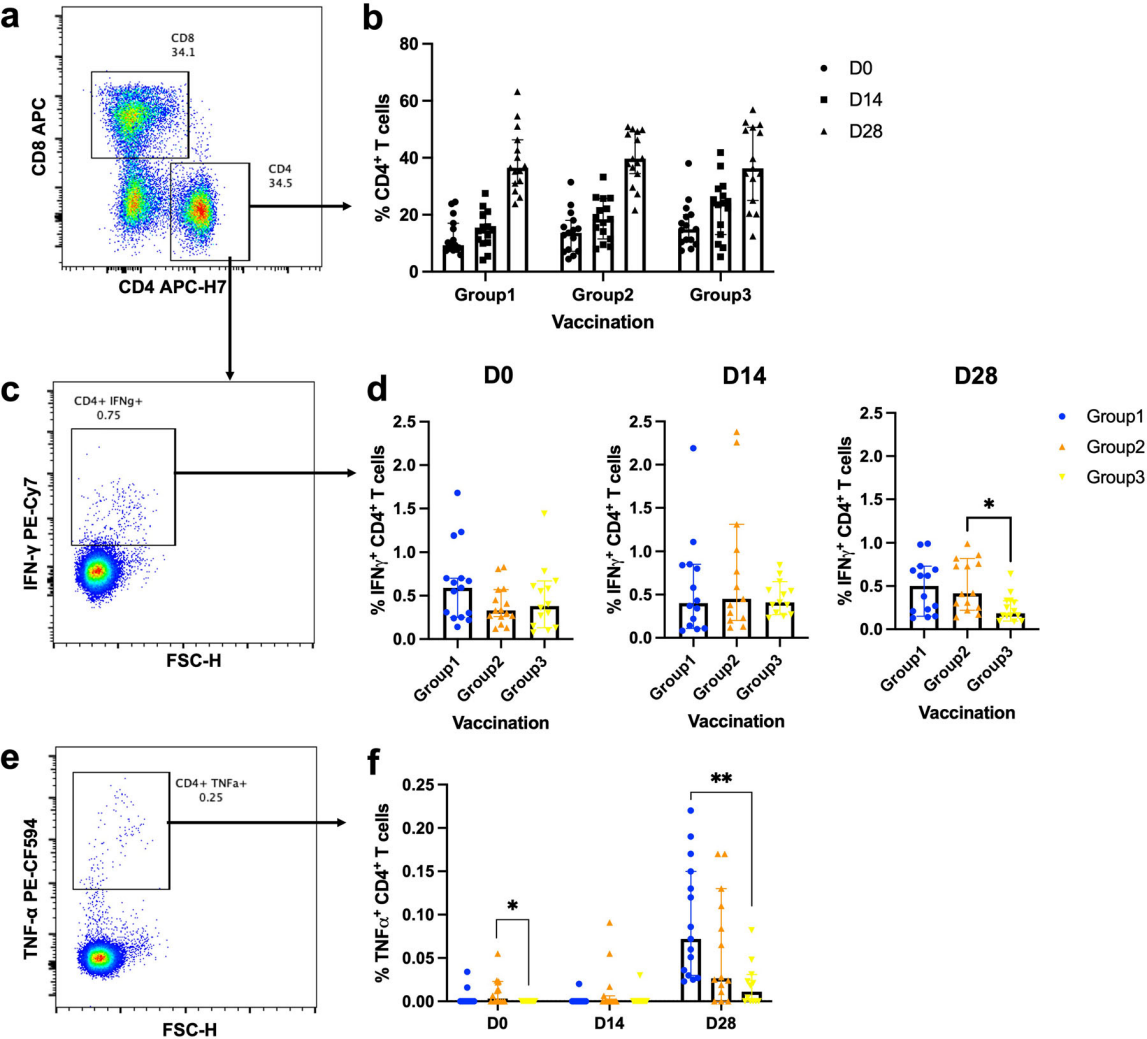
Effector cytokine production of S1-specific CD4 + T cells after boosting. All volunteers were previously completed with two doses of inactivated SARS-CoV-2 vaccines. A full dose of intramuscular viral vector booster was given at 4–8 weeks after their last vaccination (Group 1). After 4–8 weeks (Group2) and 8–12 weeks (Group3), the fractional dose of a viral vector vaccine booster was delivered intradermally. Blood was taken before (D0) and after the booster dose for 14, 28 days (D14, D28). The blood samples were processed to obtain PBMCs. The frozen PBMCs were thawed and stimulated with S1 peptide pools. The cells were stained and analysed using flow cytometry. **a** Representative flow plot shows CD8 + T cell and CD4 + T cell populations. **b** Percentage of CD4 + T cells before and after the booster dose. **c** CD4 + T cells were then selected for S1-specific IFN-γ producing cells. **d** Percentage of S1-specific IFN-γ producing CD4 + T cell responses at D0, D14 and D28. **e** Representative flow plot shows CD4 + T cells producing S1-specific TNF-α. **f** S1-specific TNF-α producing CD4 + T cells. Each symbol represents one participant presenting as a median with 95% CI (*n* = 30–34 volunteers). Statistical significance was determined using Kruskal–Wallis test, with Dunn’s multiple comparisons test between vaccinated groups. *p ≤ 0.05; **p ≤ 0.01.

To determine the effector function of T cells, their ability to secrete effector cytokines was measured. PBMCs were stimulated ex vivo with S1 peptide pools to measure viral vector boost specific responses. Surface staining was performed to identify CD8 + T cells (Fig. 6a) and CD4 + T cells (Fig. 7a). CD8 + T cell responses were comparable between study groups and at every timepoint (Fig. 6b). CD8 + T cells were intracellularly stained to measure IFN-γ and TNF-α responses (Fig. 6c, e). Before booster, S1-specific IFN-γ producing CD8 + T cells were significantly lower in the 8–12 week interval (Group3) compared to the 4–8-week interval (Group2) (*p* = 0.0413, Fig. 6d). The responses between vaccination regimens were comparable at D14 and were enhanced at D28. At D28 after boosting, the cytokine secreting CD8 + T cells of Group3 were slightly increased but the responses remained significantly less compared to intramuscular and intradermal vaccination with the interval of 4–8 weeks (*p* = 0.0003 and *p* = 0.0126 respectively, Fig. 6d). The TNF-α responses were also observed in CD8 + T cells (Fig. 6e, f). After 28 days of a booster, the TNF-α production of CD8 + T cells followed the same trend with the IFN-γ responses but the differences were not statistically significant (Fig. 6f).

CD4 + T cell responses were also analysed (Fig. 7). There was a trend for increasing CD4 + T cell responses at 14 and 28 days after boosting (Fig. 7b). S1-specific IFN-γ producing CD4 + T cells were comparable before and after 14 days of the booster. At the D28 timepoint, the intradermal vaccination could enhance the antigen-specific IFN-γ responses. However, the responses were significantly better when given at 4–8 weeks post vaccination rather than boosted at 8–12 weeks (*p* = 0.0288 respectively (Fig. 7d). The waning TNF-α responses were observed before receiving the booster dose in the longer interval compared to the shorter interval (*p* = 0.0123, Fig. 7f). ID booster with the longer interval also provided inferior TNF-α + CD4 + T cell responses compared to the conventional IM booster (*p* = 0.0011, Fig. 7f).

## Discussion

This current study demonstrated the indifferent immunogenicity; including, antibody and T cell responses between a heterologous booster with an intramuscular conventional-dose and an intradermal fractional dose of ChAdOx1 nCoV-19, for people having completed a course with two doses of the inactivated SARS-CoV-2 vaccine. Additionally, systemic adverse reactions among those receiving an intradermal fractional dose of ChAdOx1 nCoV-19 were significantly less than those receiving the intramuscular conventional-dose of ChAdOx1 nCoV-19. Unfavourable local reactions were frequently observed among those receiving an intradermal fractional dose of ChAdOx1 nCoV-19.

Compared to the previous reports of immunogenic response among the people receiving two doses of inactivated SARS-CoV-2 vaccine, this study showed the waning of antibody levels within 8 and 12 weeks^27,28^. The immunological baselines of the participants in this study were substantially low in both humoral and cellular immunities; meaning that participants in this study were at risk for both infection and severe manifestation^29^. This finding indicated the deterioration of efficacy to prevent infection and severity of the inactivated SARS-CoV-2 vaccine; thus, it endorses the concept of boosting following the conventional two doses of the inactivated SARS-CoV-2 vaccine. Within the setting in Thailand, most people were vaccinated with two doses of an inactivated SARS-CoV-2 vaccine (CoronaVac); as of June 2021, and other inactivated SARS-CoV-2 vaccines (BBIBP-CorV) as of August 2021. Hence, this waning immunity might affect the disease control preventions within this country in the last quarter^30^. The public health policy in Thailand suggests a heterogeneous booster when available vaccines are in the country (ChAdOx1 nCoV-19).

The shortage of the SARS-CoV-2 vaccine has been reported in several countries^31^. The World Health Organization (WHO) recommends distributing the vast majority of vaccines to people who have not as of yet received any vaccination^32^. At present, the number of vaccines for boosting is limited. The findings in this study served the resolution of this issue by using fractional doses (1/5) for boosting, via the intradermal technique. The immunogenicity in this study was similar to previous human studies, conducting intradermal coronavirus vaccine trials; including, DNA—ZyCoV-D (Zydus Cadila, Ahmedabad, India), INO-4800 (Inovio Pharmaceuticals, Plymouth Meeting, PA, USA) and mRNA-1273 (Moderna, Cambridge, MA, USA)^23,33,34^. While all previous studies were conducted as an initial first dose, this study fulfilled the gap of knowledge of the immunogenicity of intradermal vaccines from the perspective of a boosting dose.

The favourable findings of humoral and cellular immunogenicity among those receiving fractional intradermal vaccines are similar to several studies in rabies and viral hepatitis vaccines^35,36^. Additionally, the results of using a viral vector vaccine (ChAdOx1 nCoV-19) in this study were consistent to phase I of viral vector vaccines against malaria^22^. The well-established explanation is the abundance of dendritic cells, acting as antigen-presenting cells in the intradermal area. However, the response of local dendritic cells at the injection site can cause local reactions among those receiving fractional intradermal vaccines^22,23^. Interestingly, immediate local reaction was doubled in intradermal boosted individuals who received the booster at 8–12 weeks intervals compared to the shorter interval. This immediate reactogenicity could explain by innate immune reaction especially on Langerhans cells secreting proinflammatory cytokines^37^. The inactivated vaccines are injected intramuscularly in the deltoid so this could prime both dendritic cells and T cells to expand into resident memory cells in the skin after a few months post vaccination/antigen exposure^38,39^. Therefore, the immediate local reaction was potentially found in the latter interval. The explanation for the less systemic reaction among those receiving an intradermal vaccine is unclear, while the postulated explanation is dose-dependent associated systemic reaction^22,23^. However, no study has evaluated the immunogenicity and reactogenicity of intradermal viral vector vaccines as a booster dose.

Antigen-specific antibodies and neutralising antibody levels have been purposed as an immune correlation of protection against SARS-CoV-2. A higher level of antibodies has been observed in highly protective vaccines; such as mRNA and viral vector vaccines^25,29^. Therefore, anti-RBD-IgG were used for measuring the primary outcome of this study, which is shown as having slight differences between the conventional intramuscular booster and the fractional intradermal booster. The neutralising assays performed in preclinical and phase I studies of the current vaccines were tested against the wild-type strains^40–43^. However, the vaccine efficacy has been reported to have decreased during the breakthrough of recently mutated viruses^5^. Neutralising activity against new variants of a live virus is the closest method to predict the vaccines performance^7,29^. In the sera of vaccinated participants, the antibody neutralising function against the delta strain was significantly improved after both intradermal and intramuscular boosting. Without boosting, the neutralising function was poor, which is consistent with previous reports showing the low neutralising activity after completing two doses of the inactivated vaccine^7^. A very recent variant, Omicron, was raised as a global concern, Dejnirattisai et al. have reported neutralisation titre of Omicron by sera from vaccinees and convalescent subjects infected are substantially reduced. Following the third dose of ADZ1222, the neutralisation titers for Omicron were reduced 3.6-fold compared with Delta^44^. Therefore, we predict the neutralisation against Omicron would be reduced but would follow the same trend with our existing data against the Delta variant. Currently, the protective efficacy of the intradermal boost is being evaluated in a larger population.

As far as cellular immunities are concerned, T cell responses are used to evaluate the immunogenicity of the vaccines; especially, in preclinical studies and phase I trials^40–43,45,46^. There are several ways to measure the responses; such as ELISpot, IGRA and flow cytometry. However, the protocols are usually different, which makes it hard to compare the responses among the studies. However, it is possible to compare between treated groups in the same study^40,41,47^. In our study, we observed an increase of IFN-γ secreted T cells after boosting with an intradermal viral vector vaccine as well as the intramuscular booster. Consistent with the phase I trial of ChAdOx1 nCoV-19, strong T cell responses were observed after intramuscular injection of 2 doses as a primary vaccine^45,47^. Comparable T cell responses between intradermal and intramuscular injection were observed after immunisation with ChAd63 viral vector vaccine expressing malarial antigens^22^. In heterogenous vaccination, the memory and effector T cell responses were enhanced in the murine lungs and spleen after a prime attenuated vaccine, followed by an intradermal/intranasal boost of TB viral vector^20,48^. To observe effector T cell responses, surface and intracellular staining were analysed using flow cytometry. In consistence with our ELISpot data, cytokine secreting CD4 + T cell and CD8 + T cell responses were reduced at 28 days post booster vaccination in the 8–12 week interval compared to the shorter interval. This could explain by waning effector T cell responses before booster. S1-specific cytokine-producing CD8 + T cells were significantly decreased after 8–12 weeks post two doses of inactivated vaccines compared to 4–8 weeks post vaccination. Therefore, when these effector cells were exposed again to spike antigens from the viral vector vaccine, they could differentiate and expand less resulting in lower responses observed on D28 of the longer interval compared to the earlier interval. However, extensive T cell studies are still required concerning immune correlation with the COVID-19 vaccine efficacy.

There were several limitations in this study that should be acknowledged. The number of participants might be too low to detect common intradermal adverse reactions; such as skin necrosis. The gender proportion is biased towards the female gender. The level of the immune responses is usually different between populations, due to age, gender, race, and comorbidity^49,50^. However, the trends and the differences between vaccine regimens seem to be very much consistent. A relatively small duration for follow up, the data on infection and severity prevention is still scarce. As the inclusion criteria were limited, due to the type of vaccine, it is difficult to generalise the findings to other inactivated SARS-CoV-2 vaccines (BBIBP-CorV), or other platform vaccines. In addition, the boosting vaccine in this study only used ChAdOx1 nCoV-19; therefore, the application of other viral vector vaccines or other platform vaccines as a booster, is still unclear. This study enroled participants with a completed 2 dose of the conventional inactivated SARS-CoV-2 vaccine within 4–12 weeks; thus, the application was limited for those with longer than 12 weeks after completeness of an inactivated SARS-CoV-2 vaccine.

These data further support the ongoing evaluation of the intradermal booster of the ChAdOx1 nCoV-19 vaccine in a larger population to evaluate the booster efficacy.

## Methods

### Study procedures

This study was registered at the Thai Clinical Trials Registry (TCTR20211004001). Before enrolment, all participants gave written informed consent, and approval was obtained from the Human Research Ethics Committee (REC. 64–368–4–1). The trial was conducted according to the principles of Good Clinical Practice. Healthy adults aged 18–60 years, who had completed a two-dose regimen of inactive SARS-CoV-2 vaccine in the past 1–3 months were recruited. Key exclusion criteria were history of SARS-CoV-2 infection, uncontrolled chronic diseases, under immunosuppressive therapy, coagulation disorders, pregnancy and breastfeeding. In total, 61 participants who were in the interval of 4–8 weeks after completing vaccination were randomised using block randomisation assigned to group1 (*n* = 30), and group2 (*n* = 31). For group3, 34 participants were in the interval of >8–12 weeks.

### Sample processing

Blood samples were collected on the day of vaccination, and then on 7, 14, 28, and 90 days after the third dose. Blood samples were taken and divided into one clotted blood tube and two heparinized tubes. Samples were processed within 4–6 h of the blood draw. Clotted blood samples were processed for their serum collection. The tubes were centrifuged at 1800 r.p.m. for 10 min, and the serum was harvested for storage at −80 °C until required. Heparinized blood tubes were processed for the collection of PBMCs and plasma by density gradient centrifugation. Blood from the same participant was pooled into a 50 mL conical centrifuge tube and spun to separate blood plasma. The plasma was collected and stored at −80 °C. The remaining blood samples were diluted with RPMI (Gibco) and laid into a SepMATE tube containing Lymphoprep (STEMCELL Technologies). The samples were then centrifuged at 1200×*g* for 10 min, with the brake on. The top layer was poured into a fresh 50 mL tube and topped up with RPMI, then spun at 300×*g* for 8 min. The cell pellet was washed again with RPMI. After the last wash, the cell pellet was resuspended in 3 ml of R10 media (RPMI-1640; containing 1% penicillin-streptomycin, 2 mM l-glutamine and 10% foetal calf serum (FCS, Labtech) for counting. Cells were diluted in Trypan blue and counted using a counting chamber for use in fresh assays or for cryopreservation. All remaining cells were centrifuged (300×*g* for 8 min), and adjusted to a concentration of 3 × 10^6^ PBMCs per ml in freezing media (FCS, containing 10% DMSO). The cell suspensions were aliquoted and transferred to CoolCells (Corning) for freezing at −80 °C overnight. The tubes were then transferred into liquid nitrogen storage until required.

### Immediate and delayed adverse events

Immediate, local and systemic adverse events were monitored for 30 min after injection. Local reactions were measured as millimetres of wheal and flare; vital signs were recorded after finishing the 30-min observation. The delayed adverse events were monitored at seven days and 4 weeks after boosting, The participants were retrieved from telephone-based interviews by experienced research nurses at 7 days and completed a questionnaire regarding adverse events at 4 weeks after boosting. Delayed reactions were categorised into local and systemic reactions. Local reactions were defined as; pain, swelling, erythema or nodule at the injection site. The severity of the reactions was classified into three grades. No medication needed was grade 1, medication needed was grade 2 and a doctor’s attention required was grade 3. The rates of each adverse reaction are reported in this study.

### Quantification of SARS-CoV-2 anti-S RBD antibodies

The level of immunoglobulin class G (IgG) antibodies to the receptor-binding domain (RBD) of S1 subunit spike protein of SARS-CoV-2 were measured and quantified in human serum or plasma by using the ARCHITECT i System (Abbott, Abbott Park, Illinois, USA) chemiluminescent microparticle immunoassay (CMIA) (SARS-CoV-2 IgG II Quant, Abbott Ireland, Sligo, Ireland), with measuring reportable range from 6.8 Abbott Arbitrary Unit (AU/mL) to 80,000.0 AU/mL (up to 40,000 AU/mL with onboard 1:2 dilution). Values higher than 50 AU/mL were considered positive. Based on the evaluated dilutions of the World Health Organization (WHO) International Standard (NIBSC Code 20–136) for anti-SARS-CoV-2 human immunoglobulin in the WHO binding antibody unit (WHO BAU/mL), with the SARS-CoV-2 IgG II Quant assay with Abbott internal reference calibrators; the correlation between relationships of the AU/mL unit to the WHO BAU/mL unit is at 0.142 × AU/mL, with a 0.999 correlation coefficient.

### Plaque reduction neutralisation test (PRNT)

PRNT in this study was developed and tested by the Institute of Biological Products; a WHO- contracted laboratory at the Department of Medical Sciences. Vero cells were seeded at 2 × 10^5^ cells/well/3 ml and placed in a 37 °C, 5% CO_2_ incubator for 1 day. Test sera were initially diluted at 1:10, 1:40, 1:160 and 1:640, respectively. The SARS-CoV-2 virus was diluted in a culture medium, to yield 40–120 plaques/well in the virus control wells. Cell control wells, convalescent patient serum and normal human serum were also included as assay controls. The neutralisation was performed by mixing an equal volume of diluted serum and the optimal plaque numbers of the SARS-CoV-2 virus at 37 °C in a water bath for 1 h. After removing the culture medium from Vero cell culture plates, 200 ul of the virus-serum antibody mixture were inoculated into monolayer cells, and then rocked in the culture plates every 15 min for 1 h. Three ml of overlay semisolid medium (containing 1% of carboxymethylcellulose, Sigma-Aldrich, USA, with 1% of 10,000 units/ml Penicillin-10,000 ug/ml Streptomycin (Sigma, USA) and 10% FBS) were replaced after removing excessive viruses. All plates were incubated at 37 °C, 5% CO_2_ for 7 days. Cells were fixed with 10% (v/v) formaldehyde, then stained with 0.5% crystal violet in PBS. The number of plaques formed was counted in triplicate wells, and a percentage of plaque reduction at 50% (PRNT50) was calculated. The PRNT50 titer of the test samples is defined as the reciprocal of the highest test serum dilution, for which the virus infectivity is reduced by 50% when compared with the average plaque counts of the virus control. This was calculated by using a four-point linear regression method. Plaque counts for all serial dilutions of serum were scored to ensure that there was a dose-response.

### Ex vivo IFN-γ ELISpot assays

ELISpot assays were performed on isolated PBMCs before and after vaccination. MultiScreen-IP Filter plates (Millipore) were coated with 10 μg/mL of human anti-IFN-γ coating antibody (clone 1-D1K, Mabtech) in a carbonate buffer (Sigma-Aldrich) and stored at 4 °C overnight. The coated plates were washed three times with PBS and blocked with R10 media for a minimum of 1 h at 37 °C. After the blocking. 1.25 × 10^5^ PBMCs were added into each well as per the assigned layout. PBMCs were stimulated with a SARS-CoV-2 S1 pool (Wuhan strain) of 15-mer, with 11 amino acid overlap containing amino acid sequence 1–692 (PepTivator^®^) at a final concentration of 1 µg/mL. Each assay was performed in duplicate and incubated for 16–18 h at 37 °C with 5% CO_2_. Plates were developed by washing them six times with PBS/T, followed by the addition of 1 μg/mL of anti-IFN-γ detector antibody (7-B6-1-Biotin, Mabtech) to each well. After a 2-h incubation, plates were washed again, and 1:1,000 SA-ALP was added for 1 h at RT. After a final wash step, plates were developed using BCIP NBT-plus chromogenic substrate (Mabtech). ELISpot plates were counted using an Immunospot Microanalyzer (Cellular Technology Limited). Responses were averaged across duplicate wells, and the mean response of the unstimulated (negative control) wells were subtracted. Results are shown as SFCs/10^6^PBMCs.

### Flow cytometry analysis

Flow cytometry analysis were carried out on cryopreserved PBMCs. Cells were thawed in media containing 5 U/mL of benzonase and resuspended in complete RPMI media supplemented with 10% FCS, l-glutamine and penicillin-streptomycin (R10). Then, 1 × 10^6^ PBMC cells were seeded into a 96-well plate. The cells were washed with R10 and spun for 5 min, 470×*g* at 22 °C. Each sample was stimulated with an S1 peptide pool (ProImmune), synthesised as15-mers overlapping by ten amino acids (Supplementary Table 1). The peptide was diluted at a concentration of 2 μg/mL in R10 supplemented with anti-human CD28 and CD49d. Cells were incubated at 37 °C with 5% CO_2_ for 18 h, with GolgiPlug (BD) was added after 2 h. After the stimulation, the plates were spun and washed with PBS. Live/Dead Aqua was diluted (1:1000 in PBS; Invitrogen) and stained the cells for 10 min followed by 30 min incubation of anti-CD3, CD4 and CD8 (BD) diluted in 2% Bovine Serum Albumin (BSA) (Sigma-Aldrich) in PBS (FACS buffer) (Supplementary Table 2). After the surface staining, the cells were fixed and permeabilised using CytoFix (BD Biosciences) as per the manufacturer’s protocol. Cells were stained with anti- IFN-γ, TNF-α (BD) diluted CytoPerm buffer (BD Biosciences) for 30 min at 4 °C (Supplementary Table 2). Cells were washed with CytoPerm buffer and resuspended in FACS buffer for analysing on a CytoflexS Beckman. The acquired data were analysed using FlowJo Software (Version 10) and aged as shown in Supplementary Fig. 1.

### Statistical analysis

Statistical analyses were performed using GraphPad Prism 9 software (GraphPad Software Inc.). To determine the statistical significance, the Mann–Whitney test was used to compare two groups, while Kruskal–Wallis; followed by Dunn’s multiple comparisons test, was performed when analysing multiple groups. Values of *p* ≤ 0.05 were considered statistically significant. *p ≤ 0.05, **p ≤ 0.01, ***p ≤ 0.001, ****p ≤ 0.0001, ns non significance.

### Reporting summary

Further information on research design is available in the Nature Research Reporting Summary linked to this article.

## Supplementary information

Supplementary Table and Figures

Dataset 1

REPORTING SUMMARY

## Data Availability

All relevant data that support the findings of this study are available from the corresponding author upon reasonable request.

## References

[1] Levin, Y. et al. Ethics and execution of developing a 2nd wave COVID vaccine–Our interim phase I/II VSV-SARS-CoV2 vaccine experience. *Vaccine* **39**, 2821 (2021). 10.1016/j.vaccine.2021.04.017 PMC8043614 33896663

[2] Wendler D, Ochoa J, Millum J, Grady C, Taylor HA (2020). COVID-19 vaccine trial ethics once we have efficacious vaccines. Science.

[3] Control, E. C. f. D. P. A. Threat assessment brief: emergence of SARS CoV 2 B. 1.617 variants in India and situation in the EU/EEA (2021).

[4] Johnson, B. A. et al. Loss of furin cleavage site attenuates SARS-CoV-2 pathogenesis. *Nature*, 10.1038/s41586-021-03237-4 (2021). 10.1038/s41586-021-03237-4 PMC8175039 33494095

[5] Lopez Bernal J (2021). Effectiveness of Covid-19 vaccines against the B.1.617.2 (Delta) variant. N. Engl. J. Med..

[6] Wall EC (2021). Neutralising antibody activity against SARS-CoV-2 VOCs B.1.617.2 and B.1.351 by BNT162b2 vaccination. Lancet.

[7] Vacharathit V (2021). CoronaVac induces lower neutralising activity against variants of concern than natural infection. Lancet Infect. Dis..

[8] Garg S (2020). Hospitalization rates and characteristics of patients hospitalized with laboratory-confirmed coronavirus disease 2019 - COVID-NET, 14 states, March 1-30, 2020. Morb. Mortal. Wkly. Rep..

[9] Lustig Y (2021). BNT162b2 COVID-19 vaccine and correlates of humoral immune responses and dynamics: a prospective, single-centre, longitudinal cohort study in health-care workers. Lancet Respir. Med..

[10] Xiang T (2021). Declining levels of neutralizing antibodies against SARS-CoV-2 in convalescent COVID-19 patients one year post symptom onset. Front. Immunol..

[11] Lo Sasso, B. et al. Evaluation of anti-SARS-Cov-2 S-RBD IgG antibodies after COVID-19 mRNA BNT162b2 vaccine. *Diagnostics*10.3390/diagnostics11071135 (2021). 10.3390/diagnostics11071135 PMC8306884 34206567

[12] Tea F (2021). SARS-CoV-2 neutralizing antibodies: longevity, breadth, and evasion by emerging viral variants. PLoS Med..

[13] Liu, Z. et al. Human immunoglobulin from transchromosomic bovines hyperimmunized with SARS-CoV-2 spike antigen efficiently neutralizes viral variants. *Hum. Vaccin. Immunother.*10.1080/21645515.2021.1940652 (2021). 10.1080/21645515.2021.1940652 PMC8290372 34228597

[14] Lee, E. K., Li, Z. L., Liu, Y. K. & LeDuc, J. Strategies for vaccine prioritization and mass dispensing. *Vaccines*10.3390/vaccines9050506 (2021). 10.3390/vaccines9050506 PMC8157047 34068985

[15] Vallejos J (2021). Ivermectin to prevent hospitalizations in patients with COVID-19 (IVERCOR-COVID19) a randomized, double-blind, placebo-controlled trial. BMC Infect. Dis..

[16] Krolewiecki A (2021). Antiviral effect of high-dose ivermectin in adults with COVID-19: a proof-of-concept randomized trial. EClinicalMedicine.

[17] Migliore A, Gigliucci G, Di Marzo R, Russo D, Mammucari M (2021). Intradermal vaccination: a potential tool in the battle against the COVID-19 pandemic?. Risk Manag. Health. Policy.

[18] Schnyder JL (2020). Fractional dose of intradermal compared to intramuscular and subcutaneous vaccination - A systematic review and meta-analysis. Travel Med. Infect. Dis..

[19] Stylianou E (2015). Improvement of BCG protective efficacy with a novel chimpanzee adenovirus and a modified vaccinia Ankara virus both expressing Ag85A. Vaccine.

[20] Pinpathomrat N (2021). Using an effective TB vaccination regimen to identify immune responses associated with protection in the murine model. Vaccine.

[21] Ewer, K. J. et al. Protective CD8(+) T-cell immunity to human malaria induced by chimpanzee adenovirus-MVA immunisation. *Nat. Commun.*10.1038/ncomms3836 (2013). 10.1038/ncomms3836 PMC3868203 24284865

[22] O’Hara GA (2012). Clinical assessment of a recombinant simian adenovirus ChAd63: a potent new vaccine vector. J. Infect. Dis..

[23] Roozen GV (2022). COVID-19 vaccine dose sparing: strategies to improve vaccine equity and pandemic preparedness. Lance Glob Health.

[24] Anichini, G. et al. SARS-CoV-2 antibody response in persons with past natural infection. *N. Engl. J. Med.*10.1056/NEJMc2103825 (2021). 10.1056/NEJMc2103825 PMC8063888 33852796

[25] Feng, S. et al. Correlates of protection against symptomatic and asymptomatic SARS-CoV-2 infection. *Nat. Med.*10.1038/s41591-021-01540-1 (2021). 10.1038/s41591-021-01540-1 PMC8604724 34588689

[26] Shrotri M (2021). T cell response to SARS-CoV-2 infection in humans: a systematic review. PLoS ONE.

[27] Tanriover MD (2021). Efficacy and safety of an inactivated whole-virion SARS-CoV-2 vaccine (CoronaVac): interim results of a double-blind, randomised, placebo-controlled, phase 3 trial in Turkey. Lancet.

[28] Hitchings MDT (2021). Effectiveness of CoronaVac among healthcare workers in the setting of high SARS-CoV-2 Gamma variant transmission in Manaus, Brazil: a test-negative case-control study. Lancet Reg. Health Am..

[29] Khoury DS (2021). Neutralizing antibody levels are highly predictive of immune protection from symptomatic SARS-CoV-2 infection. Nat. Med..

[30] Department of Disease Control, Ministry of Public Health, a goverment organization. The Coronavirus disease 2019 situation. https://ddc.moph.go.th/viralpneumonia/eng/file/situation/situation-no627-270964.pdf, 2021-09-27 (2021)

[31] Henn W (2020). Allocation criteria for an initial shortage of a future SARS-CoV-2 vaccine and necessary measures for global immunity. Vaccine.

[32] Burgos, R. M. et al. The race to a COVID-19 vaccine: opportunities and challenges in development and distribution. *Drugs Context* **10**, 2020-12-2 (2021). 10.7573/dic.2020-12-2 PMC7889064 33643421

[33] Momin T (2021). Safety and Immunogenicity of a DNA SARS-CoV-2 vaccine (ZyCoV-D): results of an open-label, non-randomized phase I part of phase I/II clinical study by intradermal route in healthy subjects in India. EClinicalMedicine.

[34] Tebas P (2021). Safety and immunogenicity of INO-4800 DNA vaccine against SARS-CoV-2: a preliminary report of an open-label, Phase 1 clinical trial. EClinicalMedicine.

[35] Briggs D (2000). Antibody response of patients after postexposure rabies vaccination with small intradermal doses of purified chick embryo cell vaccine or purified Vero cell rabies vaccine. Bull. World Health Organ..

[36] Rahman F (2000). Cellular and humoral immune responses induced by intradermal or intramuscular vaccination with the major hepatitis B surface antigen. Hepatology.

[37] Gonnet J (2020). Mechanisms of innate events during skin reaction following intradermal injection of seasonal influenza vaccine. J. Proteom..

[38] Gebhardt T (2009). Memory T cells in nonlymphoid tissue that provide enhanced local immunity during infection with herpes simplex virus. Nat. Immunol..

[39] Jiang X (2012). Skin infection generates non-migratory memory CD8+ T(RM) cells providing global skin immunity. Nature.

[40] Kandeil, A. et al. Immunogenicity and safety of an inactivated SARS-CoV-2 vaccine: preclinical studies. *Vaccines*10.3390/vaccines9030214 (2021). 10.3390/vaccines9030214 PMC7999656 33802467

[41] Keech C (2020). Phase 1-2 trial of a SARS-CoV-2 recombinant spike protein nanoparticle vaccine. N. Engl. J. Med..

[42] Barrett JR (2021). Phase 1/2 trial of SARS-CoV-2 vaccine ChAdOx1 nCoV-19 with a booster dose induces multifunctional antibody responses. Nat. Med..

[43] Mulligan MJ (2020). Phase I/II study of COVID-19 RNA vaccine BNT162b1 in adults. Nature.

[44] Dejnirattisai, W. et al. SARS-CoV-2 Omicron-B.1.1.529 leads to widespread escape from neutralizing antibody responses. *Cell*10.1016/j.cell.2021.12.046 (2022). 10.1016/j.cell.2021.12.046 PMC8723827 35081335

[45] Folegatti PM (2020). Safety and immunogenicity of the ChAdOx1 nCoV-19 vaccine against SARS-CoV-2: a preliminary report of a phase 1/2, single-blind, randomised controlled trial. Lancet.

[46] van Doremalen N (2020). ChAdOx1 nCoV-19 vaccine prevents SARS-CoV-2 pneumonia in rhesus macaques. Nature.

[47] Ewer KJ (2021). T cell and antibody responses induced by a single dose of ChAdOx1 nCoV-19 (AZD1222) vaccine in a phase 1/2 clinical trial. Nat. Med..

[48] Stylianou, E. et al. Identification and evaluation of novel protective antigens for the development of a candidate TB subunit vaccine. *Infect. Immun.*10.1128/IAI.00014-18 (2018). 10.1128/IAI.00014-18 PMC6013653 29661928

[49] Ciarambino T, Para O, Giordano M (2021). Immune system and COVID-19 by sex differences and age. Women’s Health.

[50] Peckham H (2020). Male sex identified by global COVID-19 meta-analysis as a risk factor for death and ITU admission. Nat. Commun..

