# Author Correction: Immunogenicity and safety of an intradermal ChAdOx1 nCoV-19 boost in a healthy population

**DOI:** 10.1038/s41541-022-00529-2

**Published:** 2022-09-06

**Authors:** Nawamin Pinpathomrat, Porntip Intapiboon, Purilap Seepathomnarong, Jomkwan Ongarj, Ratchanon Sophonmanee, Jariya Hengprakop, Smonrapat Surasombatpattana, Supattra Uppanisakorn, Surakameth Mahasirimongkol, Waritta Sawaengdee, Supaporn Phumiamorn, Sompong Sapsutthipas, Chanon Kongkamol, Thammasin Ingviya, Pasuree Sangsupawanich, Sarunyou Chusri

**Affiliations:** 1 Department of Biomedical Sciences and Biomedical Engineering, Faculty of Medicine, Prince of Songkla University, Songkhla, Thailand; 2 Department of Internal Medicine, Faculty of Medicine, Prince of Songkla University, Songkhla, Thailand; 3 Department of Pathology, Faculty of Medicine, Prince of Songkla University, Songkhla, Thailand; 4 Clinical Research Center, Faculty of Medicine, Prince of Songkla University, Songkhla, Thailand; 5 Department of Medical Science, Ministry of Public Health, Nonthaburi, Thailand; 6 Institute of Biological Products, Department of Medical Sciences, Ministry of Public Health, Nonthaburi, Thailand; 7 Division of Digital Innovation and Data Analytics, Faculty of Medicine, Prince of Songkla University, Songkhla, Thailand

**Keywords:** Peptide vaccines, Inactivated vaccines, Viral infection, Antibodies, Viral infection

Correction to: *npj Vaccines* 10.1038/s41541-022-00475-z, published online 13 May 2022

In the original version of this Article, incorrect numbers were mistakenly inserted in Table 2, introducing a discrepancy with Fig. 2b. The incorrect numbers highlighted in bold in the below table have now been updated in the HTML and PDF version of this Article with the correct numbers. The conclusions of the Article are unchanged.

**Table 2:** Adverse events of the intramuscular (IM) and intradermal (ID) boosted ChAdOx1 nCoV-19 (Oxford-AstraZeneca; AZ) in the tested groups.

		AZ IM (4–8 wk)	AZ ID (4–8 wk)	AZ ID (8–12 wk)	
Adverse events	Total	Group1	Group2	Group3	*P* value
	*n* = 95 (%)	*n* = 30 (%)	*n* = 31 (%)	*n* = 34 (%)	
Immediate (30 min)	21 (22.1)	0 (0)	7 (22.6)	14 (41.2)	0.011
Delayed (7 days)	71 (74.7)	20 (66.7)	25 (80.6)	26 (76.5)	0.081
Pain	**43 (45.3)**	**11 (36.7)**	**16 (51.6)**	**16 (47.1)**	**0.236**
Swelling	**39 (41.1)**	**24 (80)**	**8 (25.8)**	**7 (20.6)**	<0.001
Erythema	**35 (36.8)**	**27 (90)**	**4 (12.9)**	**4 (11.8)**	<0.001
Nodule	**56 (58.9)**	**25 (83.3)**	**16 (51.6)**	**15 (44.1)**	**0.039**
Local reactions treatment					0.405
Grade 1	85 (97.7)	26 (100)	29 (100)	30 (93.8)	
Grade 2	2 (2.3)	0 (0)	0 (0)	2 (6.2)	
Systemic reactions	42 (44.2)	19 (63.3)	12 (38.7)	11 (32.4)	0.096
Fever	11 (11.6)	8 (26.7)	1 (3.2)	2 (5.9)	0.012
Chill	20 (21.1)	12 (40)	5 (16.1)	3 (8.8)	0.073
Fatigue	24 (25.3)	11 (36.7)	5 (16.1)	8 (23.5)	0.126
Headache	22 (23.2)	11 (36.7)	6 (19.4)	5 (14.7)	0.222
Myalgia	31 (32.6)	16 (53.3)	9 (29)	6 (17.6)	0.095
Dyspnea	4 (4.2)	3 (10)	1 (3.2)	0 (0)	0.354
Joint pain	4 (4.2)	3 (10)	1 (3.2)	0 (0)	0.354
Vomiting	1 (1.1)	1 (3.3)	0 (0)	0 (0)	0.492
Systemic reactions treatment					0.174
Grade 1	14 (33.3)	2 (10.5)	4 (33.3)	8 (72.7)	
Grade 2	28 (66.7)	17 (89.5)	8 (66.7)	3 (27.3)	

*P* value refers comparisons between Group1 and Group2 which were determined using Chi’s square test.

