# Supplementary material for: Immunogenicity and safety of an intradermal ChAdOx1 nCoV-19 boost in a healthy population
Source: NPJ Vaccines. 2022 May 13;7:52. doi: 10.1038/s41541-022-00475-z (PMC9106663; doi:10.1038/s41541-022-00475-z)
Supplement: Supplementary file 1 — Supplementary Table and Figures [file 41541_2022_475_MOESM1_ESM.pdf]

Supplementary

Supplementary Table 1. Amino acid sequences of SARS-CoV-2 S1 peptides (ProImmune).

Peptide number	Amino acid start position	Sequence
1	1	MFVFLVLLPLVSSQC
2	6	VLLPLVSSQCVNLTT
3	11	VSSQCVNLTTRTQLP
4	16	VNLTTRTQLPPAYTN
5	21	RTQLPPAYTNSFTRG
6	26	PAYTNSFTRGVYYPD
7	31	SFTRGVYYPDKVFRS
8	36	VYYPDKVFRSSVLHS
9	41	KVFRSSVLHSTQDLF
10	46	SVLHSTQDLFLPFFS
11	51	TQDLFLPFFSNVTWF
12	56	LPFFSNVTWFHAIHV
13	61	NVTWFHAIHVSGTNG
14	66	HAIHVSGTNGTKRFD
15	71	SGTNGTKRFDNPVLP
16	76	TKRFDNPVLPFNDGV
17	81	NPVLPFNDGVYFAST
18	86	FNDGVYFASTEKSNI
19	91	YFASTEKSNIIRGWI
20	96	EKSNIIRGWIFGTTL
21	101	IRGWIFGTTLDSKTQ
22	106	FGTTLDSKTQSLLIV
23	111	DSKTQSLLIVNNATN
24	116	SLLIVNNATNVVIKV
25	121	NNATNVVIKVCEFQF
26	126	VVIKVCEFQFCNDPF
27	131	CEFQFCNDPFLGVYY
28	136	CNDPFLGVYYHKNNK
29	141	LGVYYHKNNKSWMES
30	146	HKNNKSWMESEFRVY
31	151	SWMESEFRVYSSANN
32	156	EFRVYSSANNCTFEY

Peptide number	Amino acid start position	Sequence
33	161	SSANNCTFEYVSQPF
34	166	CTFEYVSQPFLMDLE
35	171	VSQPFLMDLEGKQGN
36	176	LMDLEGKQGNFKNLR
37	181	GKQGNFKNLREFVFK
38	186	FKNLREFVFKNIDGY
39	191	EFVFKNIDGYFKIYS
40	196	NIDGYFKIYSKHTPI
41	201	FKIYSKHTPINLVRD
42	206	KHTPINLVRDLPQGF
43	211	NLVRDLPQGFSALEP
44	216	LPQGFSALEPLVDLP
45	221	SALEPLVDLPIGINI
46	226	LVDLPIGINITRFQT
47	231	IGINITRFQTLLALH
48	236	TRFQTLLALHRSYLT
49	241	LLALHRSYLTPGDSS
50	246	RSYLTPGDSSSGWTA
51	251	PGDSSSGWTAGAAAY
52	256	SGWTAGAAAYYVGYL
53	261	GAAAYYVGYLQPRTF
54	266	YVGYLQPRTFLLKYN
55	271	QPRTFLLKYNENGTI
56	276	LLKYNENGTITDAVD
57	281	ENGTITDAVDCALDP
58	286	TDAVDCALDPLSETK
59	291	CALDPLSETKCTLKS
60	296	LSETKCTLKSFTVEK
61	301	CTLKSFTVEKGIYQT
62	306	FTVEKGIYQTSNFRV
63	311	GIYQTSNFRVQPTES
64	316	SNFRVQPTESIVRFP
65	321	QPTESIVRFPNITNL
66	326	IVRFPNITNLCPFGE
67	331	NITNLCPFGEVFNAT
68	336	CPFGEVFNATRFASV
69	341	VFNATRFASVYAWNR

Peptide number	Amino acid start position	Sequence
70	346	RFASVYAWNRKRISN
71	351	YAWNRKRISNCVADY
72	356	KRISNCVADYSVLYN
73	361	CVADYSVLYNSASFS
74	366	SVLYNSASFSTFKCY
75	371	SASFSTFKCYGVSPT
76	376	TFKCYGVSPTKLNDL
77	381	GVSPTKLNDLCFTNV
78	386	KLNDLCFTNVYADSF
79	391	CFTNVYADSFVIRGD
80	396	YADSFVIRGDEVRQI
81	401	VIRGDEVRQIAPGQT
82	406	EVRQIAPGQTGKIAD
83	411	APGQTGKIADYNYKL
84	416	GKIADYNYKLPDDFT
85	421	YNYKLPDDFTGCVIA
86	426	PDDFTGCVIAWNSNN
87	431	GCVIAWNSNNLDSKV
88	436	WNSNNLDSKVGGNYN
89	441	LDSKVGGNYNYLYRL
90	446	GGNYNYLYRLFRKSN
91	451	YLYRLFRKSNLKPFE
92	456	FRKSNLKPFERDIST
93	461	LKPFERDISTEIYQA
94	466	RDISTEIYQAGSTPC
95	471	EIYQAGSTPCNGVEG
96	476	GSTPCNGVEGFNCYF
97	481	NGVEGFNCYFPLQSY
98	486	FNCYFPLQSYGFQPT
99	491	PLQSYGFQPTNGVGY
100	496	GFQPTNGVGYQPYRV
101	501	NGVGYQPYRVVVLSF
102	506	QPYRVVVLSFELLHA
103	511	VVLSFELLHAPATVC
104	516	ELLHAPATVCGPKKS
105	521	PATVCGPKKSTNLVK
106	526	GPKKSTNLVKNKCVN

Peptide number	Amino acid start position	Sequence
107	531	TNLVKNKCVNFNFNG
108	536	NKCVNFNFNGLTGTG
109	541	FNFNGLTGTGVLTES
110	546	LTGTGVLTESNKKFL
111	551	VLTESNKKFLPFQQF
112	556	NKKFLPFQQFGRDIA
113	561	PFQQFGRDIADTTDA
114	566	GRDIADTTDAVRDPQ
115	571	DTTDAVRDPQTLEIL
116	576	VRDPQTLEILDITPC
117	581	TLEILDITPCSFGGV
118	586	DITPCSFGGVSVITP
119	591	SFGGVSVITPGTNTS
120	596	SVITPGTNTSNQVAV
121	601	GTNTSNQVAVLYQDV
122	606	NQVAVLYQDVNCTEV
123	611	LYQDVNCTEVPVAIH
124	616	NCTEVPVAIHADQLT
125	621	PVAIHADQLTPTWRV
126	626	ADQLTPTWRVYSTGS
127	631	PTWRVYSTGSNVFQT
128	636	YSTGSNVFQTRAGCL
129	641	NVFQTRAGCLIGAEH
130	646	RAGCLIGAEHVNNSY
131	651	IGAEHVNNSYECDIP
132	656	VNNSYECDIPIGAGI
133	661	ECDIPIGAGICASYQ
134	666	IGAGICASYQTQTNS

Supplementary Table 2. Fluorochrome conjugated antibodies for flow cytometry analysis.

Antibody	Common Fluorochrome	Dilution	Clone	Source
CD3	PerCP	1:50	SP34-2	BD Horizon
CD4	APC-H7	1:200	SK3	BD
CD8	APC	1:200	SK1	BD
L/D	Aqua	1:1000	-	Invitrogen
INF-Gamma	PE-Cy7	1:100	B27	BD Pharmingen
TNF-alpha	PE-CF594	1:100	MAb11	BD Horizon

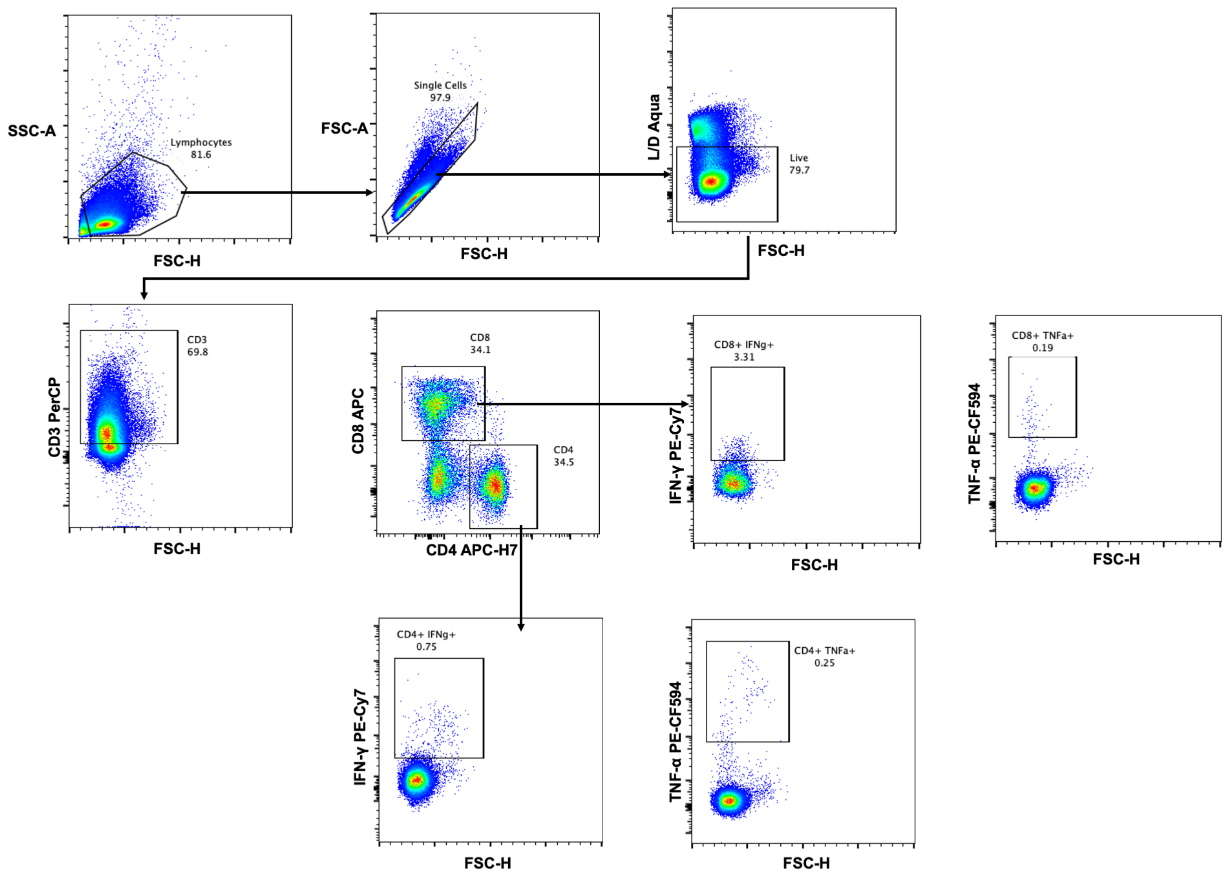

Supplementary Figure 1. Gating strategy for the effector cytokine production of T cells after boosting
